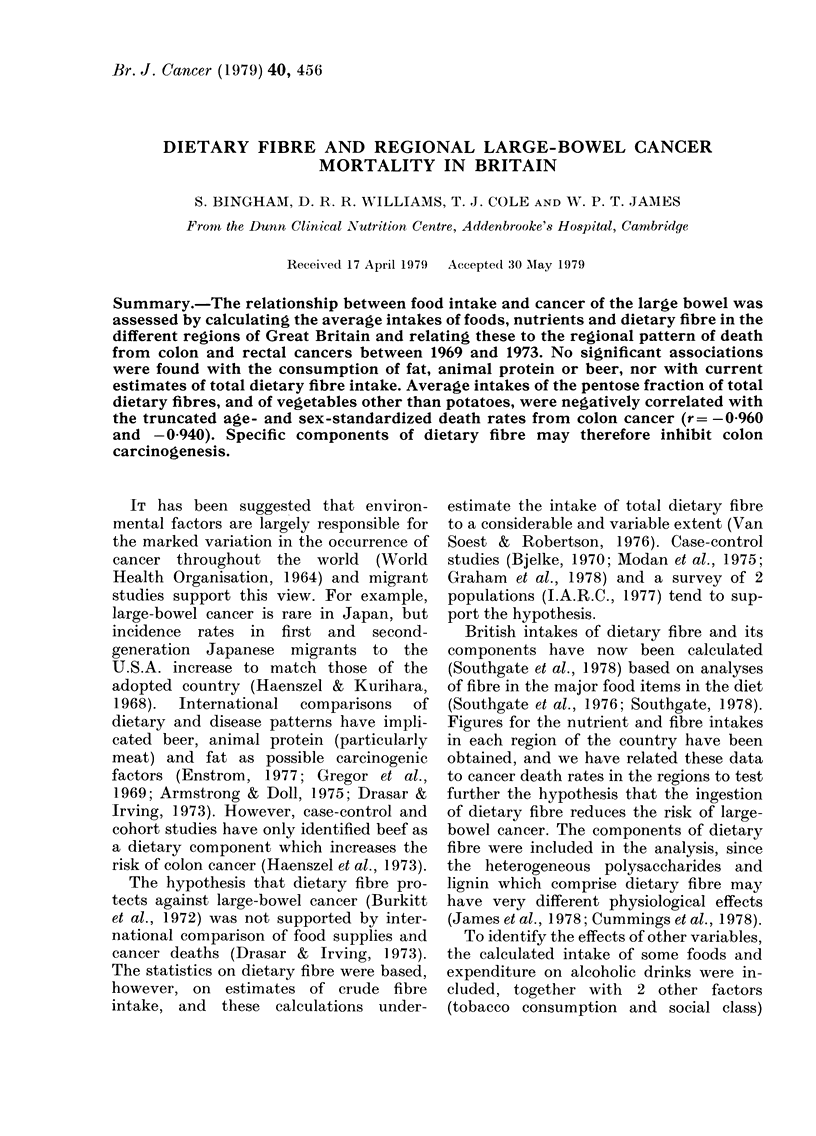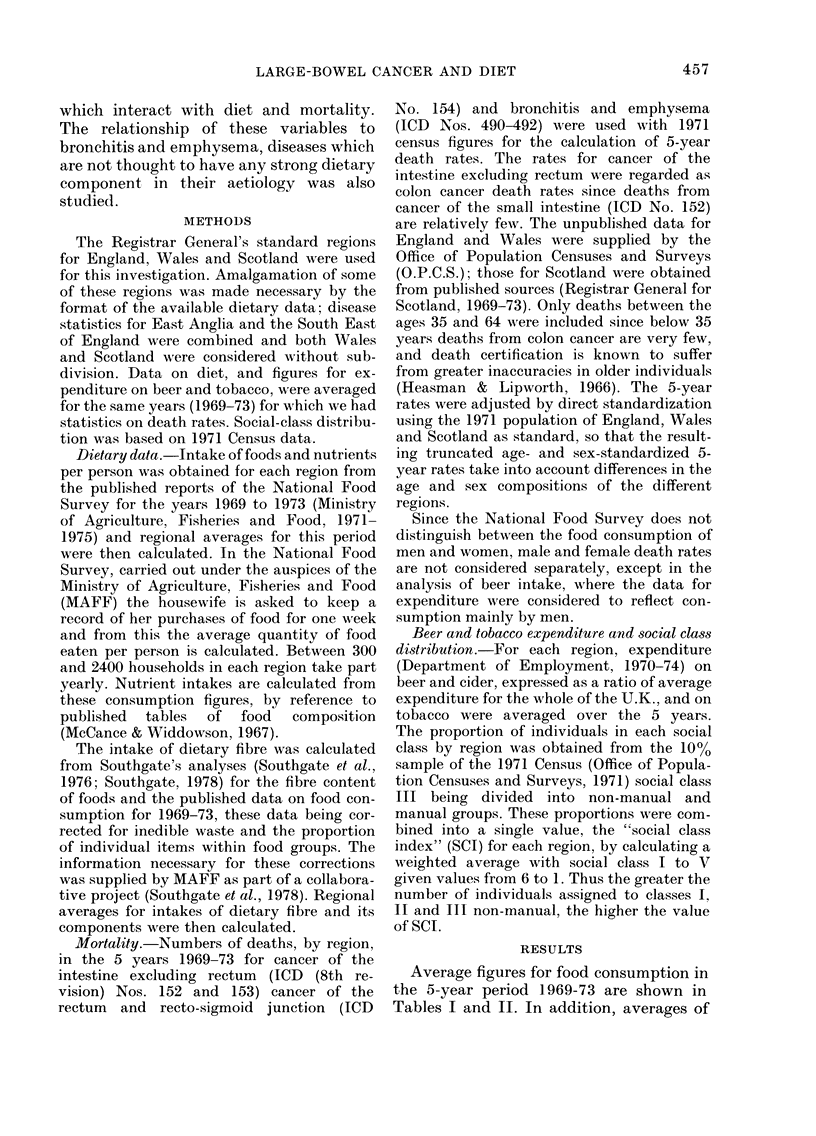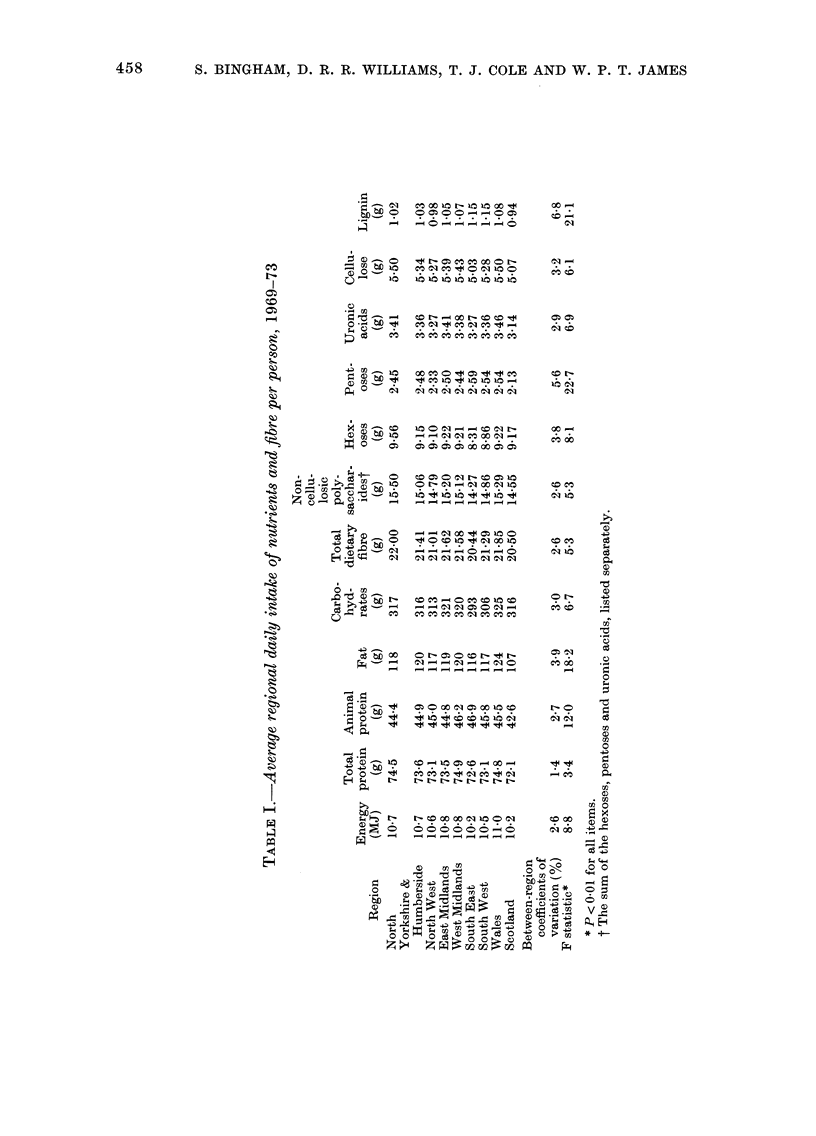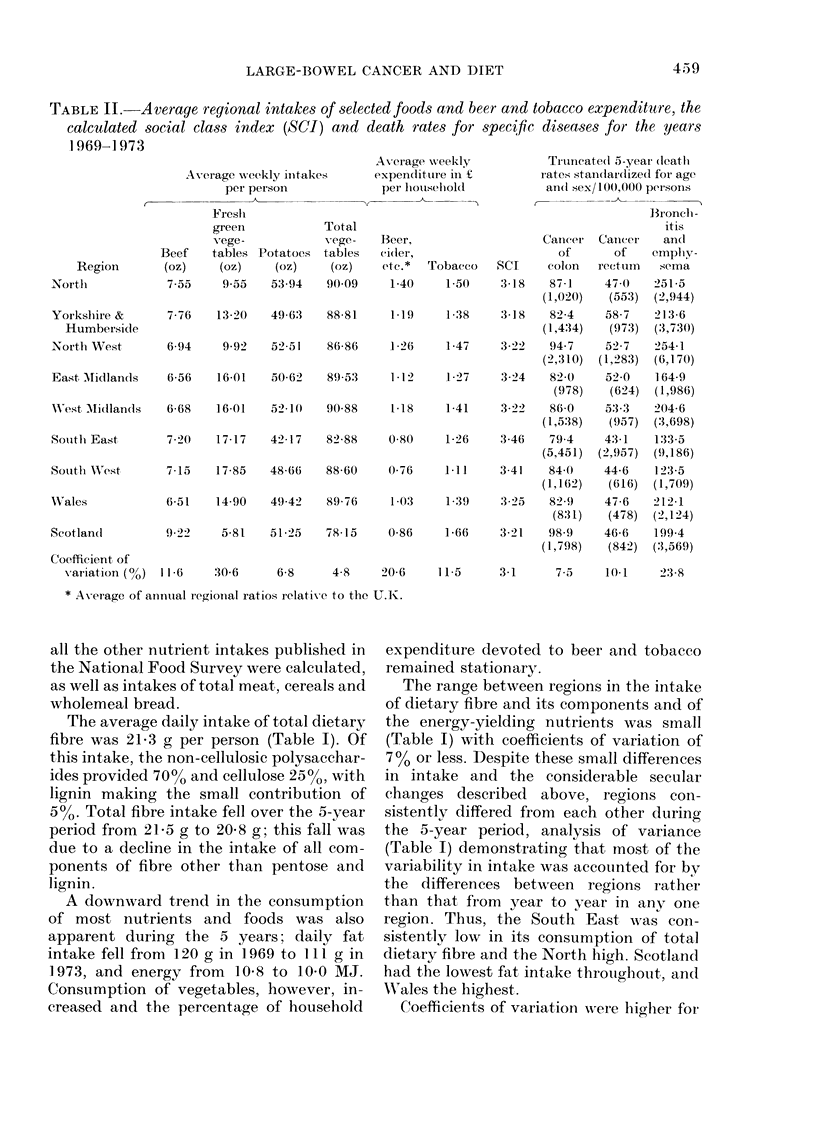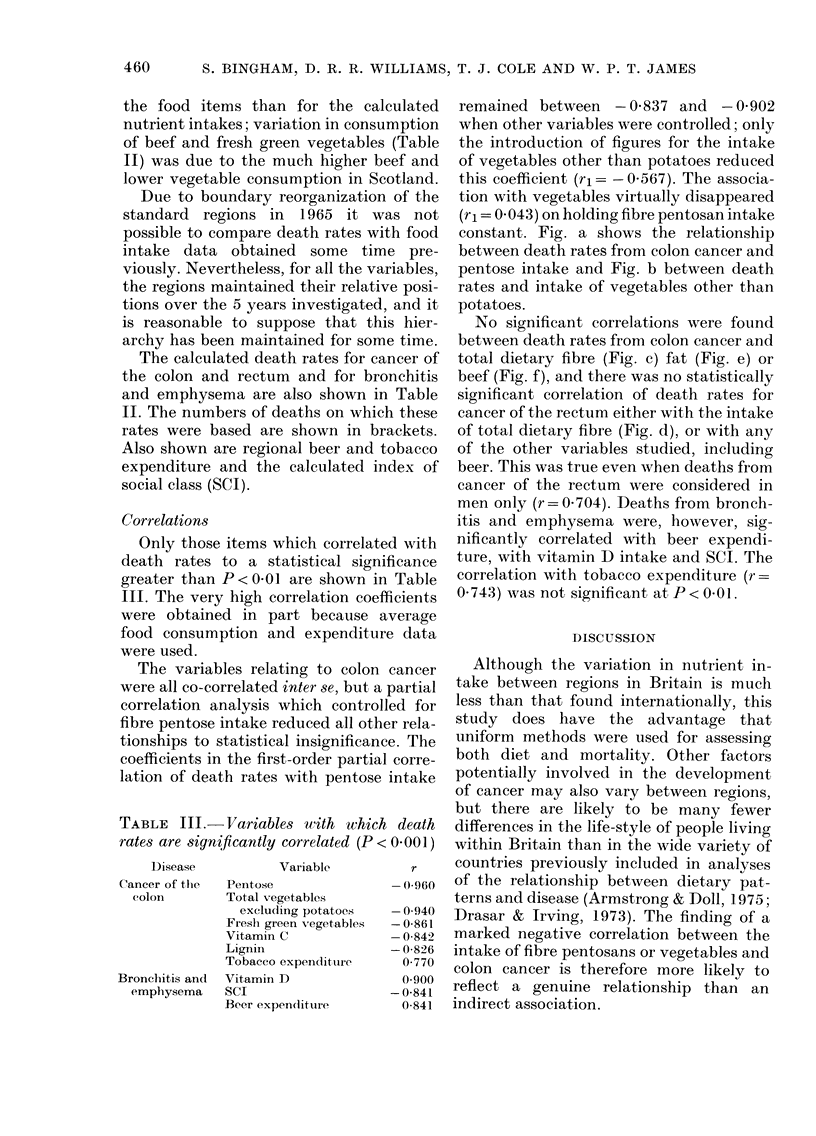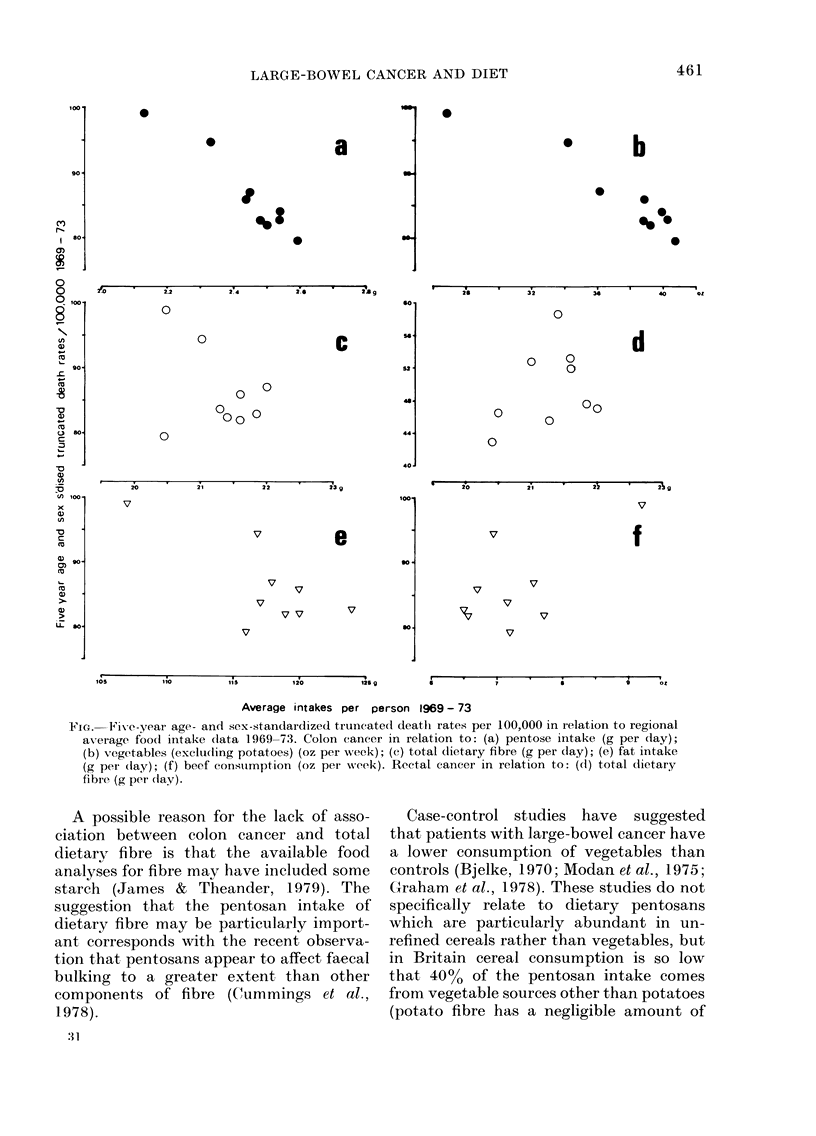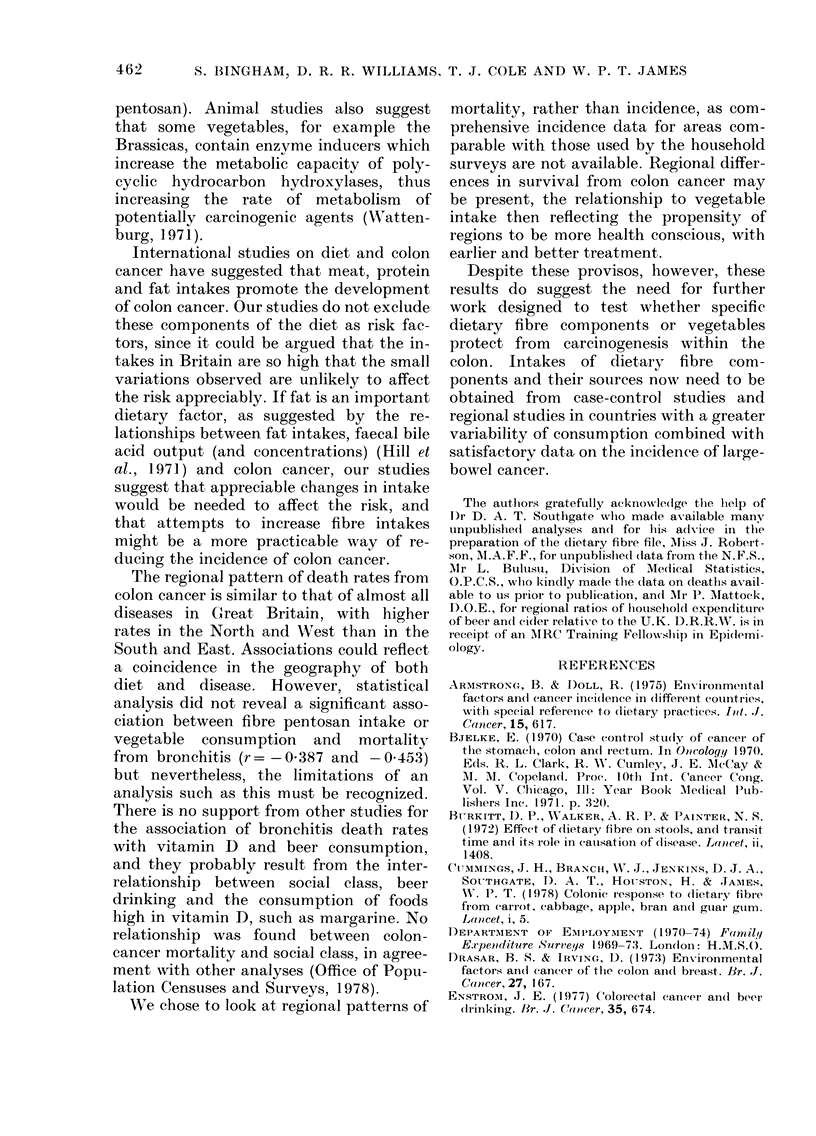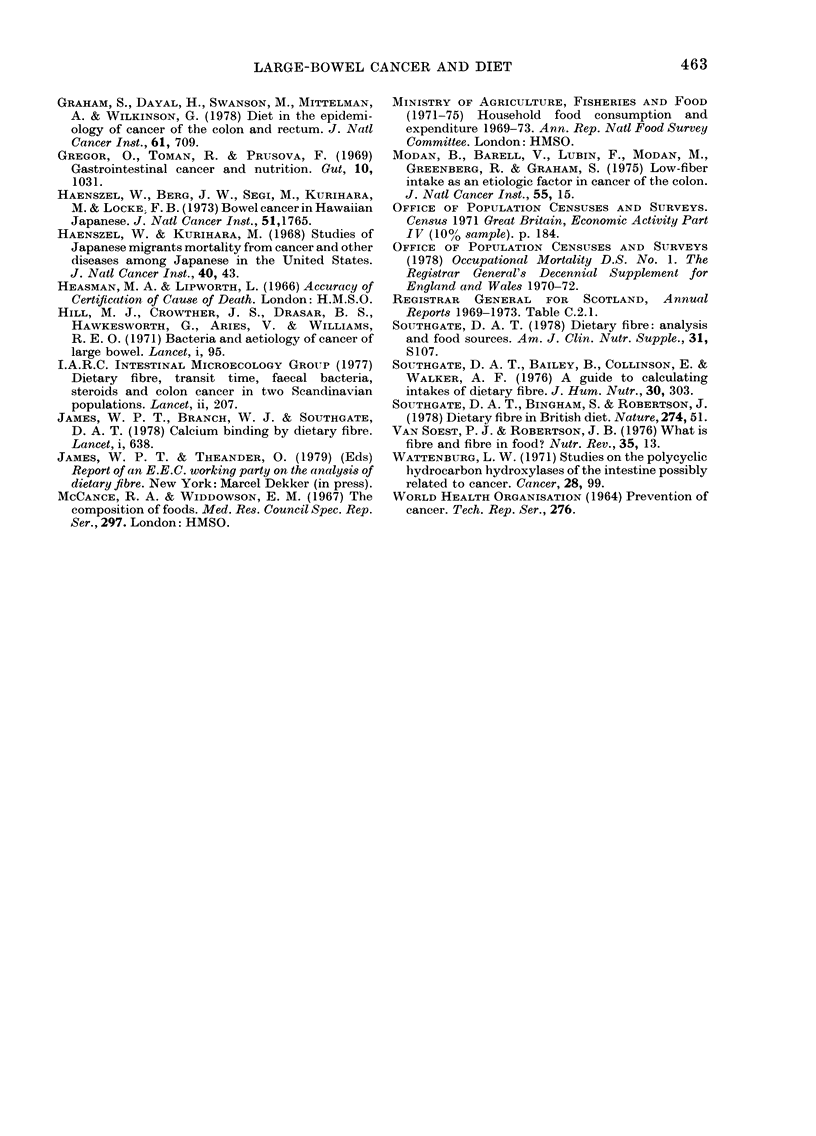# Dietary fibre and regional large-bowel cancer mortality in Britain.

**DOI:** 10.1038/bjc.1979.201

**Published:** 1979-09

**Authors:** S. Bingham, D. R. Williams, T. J. Cole, W. P. James

## Abstract

The relationship between food intake and cancer of the large bowel was assessed by calculating the average intakes of foods, nutrients and dietary fibre in the different regions of Great Britain and relating these to the regional pattern of death from colon and rectal cancers between 1969 and 1973. No significant associations were found with the consumption of fat, animal protein or beer, nor with current estimates of total dietary fibre intake. Average intakes of the pentose fraction of total dietary fibres, and of vegetables other than potatoes, were negatively correlated with the truncated age- and sex-standardized death rates from colon cancer (r = -0.960 and -0.940). Specific components of dietary fibre may therefore inhibit colon carcinogenesis.


*Br. J. Cancer* (1979) **40,** 456

### DIETARY FIBRE AND REGIONAL LARGE-BOWEL CANCER MORTALITY IN BRITAIN

S. BINGHAM, D. R. R. WILLIAMS, T. J. COLE AND W. P. T. JAMES

*From the Dunn Clinical Nutrition Centre, Addenbrooke's Hospital, Cambridge*

Received 17 April 1979 Accepted 30 May 1979

**Summary.—The relationship between food intake and cancer of the large bowel was assessed by calculating the average intakes of foods, nutrients and dietary fibre in the different regions of Great Britain and relating these to the regional pattern of death from colon and rectal cancers between 1969 and 1973. No significant associations were found with the consumption of fat, animal protein or beer, nor with current estimates of total dietary fibre intake. Average intakes of the pentose fraction of total dietary fibres, and of vegetables other than potatoes, were negatively correlated with the truncated age- and sex-standardized death rates from colon cancer ($r = -0{\cdot}960$ and $-0{\cdot}940$). Specific components of dietary fibre may therefore inhibit colon carcinogenesis.**

IT has been suggested that environmental factors are largely responsible for the marked variation in the occurrence of cancer throughout the world (World Health Organisation, 1964) and migrant studies support this view. For example, large-bowel cancer is rare in Japan, but incidence rates in first and second-generation Japanese migrants to the U.S.A. increase to match those of the adopted country (Haenszel & Kurihara, 1968). International comparisons of dietary and disease patterns have implicated beer, animal protein (particularly meat) and fat as possible carcinogenic factors (Enstrom, 1977; Gregor *et al.*, 1969; Armstrong & Doll, 1975; Drasar & Irving, 1973). However, case-control and cohort studies have only identified beef as a dietary component which increases the risk of colon cancer (Haenszel *et al.*, 1973).

The hypothesis that dietary fibre protects against large-bowel cancer (Burkitt *et al.*, 1972) was not supported by international comparison of food supplies and cancer deaths (Drasar & Irving, 1973). The statistics on dietary fibre were based, however, on estimates of crude fibre intake, and these calculations underestimate the intake of total dietary fibre to a considerable and variable extent (Van Soest & Robertson, 1976). Case-control studies (Bjelke, 1970; Modan *et al.*, 1975; Graham *et al.*, 1978) and a survey of 2 populations (I.A.R.C., 1977) tend to support the hypothesis.

British intakes of dietary fibre and its components have now been calculated (Southgate *et al.*, 1978) based on analyses of fibre in the major food items in the diet (Southgate *et al.*, 1976; Southgate, 1978). Figures for the nutrient and fibre intakes in each region of the country have been obtained, and we have related these data to cancer death rates in the regions to test further the hypothesis that the ingestion of dietary fibre reduces the risk of large-bowel cancer. The components of dietary fibre were included in the analysis, since the heterogeneous polysaccharides and lignin which comprise dietary fibre may have very different physiological effects (James *et al.*, 1978; Cummings *et al.*, 1978).

To identify the effects of other variables, the calculated intake of some foods and expenditure on alcoholic drinks were included, together with 2 other factors (tobacco consumption and social class)

which interact with diet and mortality. The relationship of these variables to bronchitis and emphysema, diseases which are not thought to have any strong dietary component in their aetiology was also studied.

### METHODS

The Registrar General's standard regions for England, Wales and Scotland were used for this investigation. Amalgamation of some of these regions was made necessary by the format of the available dietary data; disease statistics for East Anglia and the South East of England were combined and both Wales and Scotland were considered without subdivision. Data on diet, and figures for expenditure on beer and tobacco, were averaged for the same years (1969–73) for which we had statistics on death rates. Social-class distribution was based on 1971 Census data.

*Dietary data.*—Intake of foods and nutrients per person was obtained for each region from the published reports of the National Food Survey for the years 1969 to 1973 (Ministry of Agriculture, Fisheries and Food, 1971–1975) and regional averages for this period were then calculated. In the National Food Survey, carried out under the auspices of the Ministry of Agriculture, Fisheries and Food (MAFF) the housewife is asked to keep a record of her purchases of food for one week and from this the average quantity of food eaten per person is calculated. Between 300 and 2400 households in each region take part yearly. Nutrient intakes are calculated from these consumption figures, by reference to published tables of food composition (McCance & Widdowson, 1967).

The intake of dietary fibre was calculated from Southgate's analyses (Southgate *et al.*, 1976; Southgate, 1978) for the fibre content of foods and the published data on food consumption for 1969–73, these data being corrected for inedible waste and the proportion of individual items within food groups. The information necessary for these corrections was supplied by MAFF as part of a collaborative project (Southgate *et al.*, 1978). Regional averages for intakes of dietary fibre and its components were then calculated.

*Mortality.*—Numbers of deaths, by region, in the 5 years 1969–73 for cancer of the intestine excluding rectum (ICD (8th revision) Nos. 152 and 153) cancer of the rectum and recto-sigmoid junction (ICD No. 154) and bronchitis and emphysema (ICD Nos. 490–492) were used with 1971 census figures for the calculation of 5-year death rates. The rates for cancer of the intestine excluding rectum were regarded as colon cancer death rates since deaths from cancer of the small intestine (ICD No. 152) are relatively few. The unpublished data for England and Wales were supplied by the Office of Population Censuses and Surveys (O.P.C.S.); those for Scotland were obtained from published sources (Registrar General for Scotland, 1969–73). Only deaths between the ages 35 and 64 were included since below 35 years deaths from colon cancer are very few, and death certification is known to suffer from greater inaccuracies in older individuals (Heasman & Lipworth, 1966). The 5-year rates were adjusted by direct standardization using the 1971 population of England, Wales and Scotland as standard, so that the resulting truncated age- and sex-standardized 5-year rates take into account differences in the age and sex compositions of the different regions.

Since the National Food Survey does not distinguish between the food consumption of men and women, male and female death rates are not considered separately, except in the analysis of beer intake, where the data for expenditure were considered to reflect consumption mainly by men.

*Beer and tobacco expenditure and social class distribution.*—For each region, expenditure (Department of Employment, 1970–74) on beer and cider, expressed as a ratio of average expenditure for the whole of the U.K., and on tobacco were averaged over the 5 years. The proportion of individuals in each social class by region was obtained from the 10% sample of the 1971 Census (Office of Population Censuses and Surveys, 1971) social class III being divided into non-manual and manual groups. These proportions were combined into a single value, the "social class index" (SCI) for each region, by calculating a weighted average with social class I to V given values from 6 to 1. Thus the greater the number of individuals assigned to classes I, II and III non-manual, the higher the value of SCI.

### RESULTS

Average figures for food consumption in the 5-year period 1969-73 are shown in Tables I and II. In addition, averages of

TABLE I.—*Average regional daily intake of nutrients and fibre per person*, 1969–73

| Region | Energy (MJ) | Total protein (g) | Animal protein (g) | Fat (g) | Carbo-hyd-rates (g) | Total dietary fibre (g) | Non-cellu-losic poly-sacchar-ides† (g) | Hex-oses (g) | Pent-oses (g) | Uronic acids (g) | Cellu-lose (g) | Lignin (g) |
|---|---|---|---|---|---|---|---|---|---|---|---|---|
| North | 10·7 | 74·5 | 44·4 | 118 | 317 | 22·00 | 15·50 | 9·56 | 2·45 | 3·41 | 5·50 | 1·02 |
| Yorkshire & Humberside | 10·7 | 73·6 | 44·9 | 120 | 316 | 21·41 | 15·06 | 9·15 | 2·48 | 3·36 | 5·34 | 1·03 |
| North West | 10·6 | 73·1 | 45·0 | 117 | 313 | 21·01 | 14·79 | 9·10 | 2·33 | 3·27 | 5·27 | 0·98 |
| East Midlands | 10·8 | 73·5 | 44·8 | 119 | 321 | 21·62 | 15·20 | 9·22 | 2·50 | 3·41 | 5·39 | 1·05 |
| West Midlands | 10·8 | 74·9 | 46·2 | 120 | 320 | 21·58 | 15·12 | 9·21 | 2·44 | 3·38 | 5·43 | 1·07 |
| South East | 10·2 | 72·6 | 46·9 | 116 | 293 | 20·44 | 14·27 | 8·31 | 2·59 | 3·27 | 5·03 | 1·15 |
| South West | 10·5 | 73·1 | 45·8 | 117 | 306 | 21·29 | 14·86 | 8·86 | 2·54 | 3·36 | 5·28 | 1·15 |
| Wales | 11·0 | 74·8 | 45·5 | 124 | 325 | 21·85 | 15·29 | 9·22 | 2·54 | 3·46 | 5·50 | 1·08 |
| Scotland | 10·2 | 72·1 | 42·6 | 107 | 316 | 20·50 | 14·55 | 9·17 | 2·13 | 3·14 | 5·07 | 0·94 |
| Between-region coefficients of variation (%) | 2·6 | 1·4 | 2·7 | 3·9 | 3·0 | 2·6 | 2·6 | 3·8 | 5·6 | 2·9 | 3·2 | 6·8 |
| F statistic* | 8·8 | 3·4 | 12·0 | 18·2 | 6·7 | 5·3 | 5·3 | 8·1 | 22·7 | 6·9 | 6·1 | 21·1 |

* $P < 0{\cdot}01$ for all items.
† The sum of the hexoses, pentoses and uronic acids, listed separately.

TABLE II.—*Average regional intakes of selected foods and beer and tobacco expenditure, the calculated social class index (SCI) and death rates for specific diseases for the years* 1969–1973

| | Average weekly intakes per person | | | | Average weekly expenditure in £ per household | | | Truncated 5-year death rates standardized for age and sex/100,000 persons | | |
|---|---|---|---|---|---|---|---|---|---|---|
| Region | Beef (oz) | Fresh green vegetables (oz) | Potatoes (oz) | Total vegetables (oz) | Beer, cider, etc.* | Tobacco | SCI | Cancer of colon | Cancer of rectum | Bronchitis and emphysema |
| North | 7·55 | 9·55 | 53·94 | 90·09 | 1·40 | 1·50 | 3·18 | 87·1 (1,020) | 47·0 (553) | 251·5 (2,944) |
| Yorkshire & Humberside | 7·76 | 13·20 | 49·63 | 88·81 | 1·19 | 1·38 | 3·18 | 82·4 (1,434) | 58·7 (973) | 213·6 (3,730) |
| North West | 6·94 | 9·92 | 52·51 | 86·86 | 1·26 | 1·47 | 3·22 | 94·7 (2,310) | 52·7 (1,283) | 254·1 (6,170) |
| East Midlands | 6·56 | 16·01 | 50·62 | 89·53 | 1·12 | 1·27 | 3·24 | 82·0 (978) | 52·0 (624) | 164·9 (1,986) |
| West Midlands | 6·68 | 16·01 | 52·10 | 90·88 | 1·18 | 1·41 | 3·22 | 86·0 (1,538) | 53·3 (957) | 204·6 (3,698) |
| South East | 7·20 | 17·17 | 42·17 | 82·88 | 0·80 | 1·26 | 3·46 | 79·4 (5,451) | 43·1 (2,957) | 133·5 (9,186) |
| South West | 7·15 | 17·85 | 48·66 | 88·60 | 0·76 | 1·11 | 3·41 | 84·0 (1,162) | 44·6 (616) | 123·5 (1,709) |
| Wales | 6·51 | 14·90 | 49·42 | 89·76 | 1·03 | 1·39 | 3·25 | 82·9 (831) | 47·6 (478) | 212·1 (2,124) |
| Scotland | 9·22 | 5·81 | 51·25 | 78·15 | 0·86 | 1·66 | 3·21 | 98·9 (1,798) | 46·6 (842) | 199·4 (3,569) |
| Coefficient of variation (%) | 11·6 | 30·6 | 6·8 | 4·8 | 20·6 | 11·5 | 3·1 | 7·5 | 10·1 | 23·8 |

* Average of annual regional ratios relative to the U.K.

all the other nutrient intakes published in the National Food Survey were calculated, as well as intakes of total meat, cereals and wholemeal bread.

The average daily intake of total dietary fibre was 21·3 g per person (Table I). Of this intake, the non-cellulosic polysaccharides provided 70% and cellulose 25%, with lignin making the small contribution of 5%. Total fibre intake fell over the 5-year period from 21·5 g to 20·8 g; this fall was due to a decline in the intake of all components of fibre other than pentose and lignin.

A downward trend in the consumption of most nutrients and foods was also apparent during the 5 years; daily fat intake fell from 120 g in 1969 to 111 g in 1973, and energy from 10·8 to 10·0 MJ. Consumption of vegetables, however, increased and the percentage of household expenditure devoted to beer and tobacco remained stationary.

The range between regions in the intake of dietary fibre and its components and of the energy-yielding nutrients was small (Table I) with coefficients of variation of 7% or less. Despite these small differences in intake and the considerable secular changes described above, regions consistently differed from each other during the 5-year period, analysis of variance (Table I) demonstrating that most of the variability in intake was accounted for by the differences between regions rather than that from year to year in any one region. Thus, the South East was consistently low in its consumption of total dietary fibre and the North high. Scotland had the lowest fat intake throughout, and Wales the highest.

Coefficients of variation were higher for the food items than for the calculated nutrient intakes; variation in consumption of beef and fresh green vegetables (Table II) was due to the much higher beef and lower vegetable consumption in Scotland.

Due to boundary reorganization of the standard regions in 1965 it was not possible to compare death rates with food intake data obtained some time previously. Nevertheless, for all the variables, the regions maintained their relative positions over the 5 years investigated, and it is reasonable to suppose that this hierarchy has been maintained for some time.

The calculated death rates for cancer of the colon and rectum and for bronchitis and emphysema are also shown in Table II. The numbers of deaths on which these rates were based are shown in brackets. Also shown are regional beer and tobacco expenditure and the calculated index of social class (SCI).

*Correlations*

Only those items which correlated with death rates to a statistical significance greater than $P < 0{\cdot}01$ are shown in Table III. The very high correlation coefficients were obtained in part because average food consumption and expenditure data were used.

The variables relating to colon cancer were all co-correlated *inter se*, but a partial correlation analysis which controlled for fibre pentose intake reduced all other relationships to statistical insignificance. The coefficients in the first-order partial correlation of death rates with pentose intake remained between $-0{\cdot}837$ and $-0{\cdot}902$ when other variables were controlled; only the introduction of figures for the intake of vegetables other than potatoes reduced this coefficient ($r_1 = -0{\cdot}567$). The association with vegetables virtually disappeared ($r_1 = 0{\cdot}043$) on holding fibre pentosan intake constant. Fig. a shows the relationship between death rates from colon cancer and pentose intake and Fig. b between death rates and intake of vegetables other than potatoes.

No significant correlations were found between death rates from colon cancer and total dietary fibre (Fig. c) fat (Fig. e) or beef (Fig. f), and there was no statistically significant correlation of death rates for cancer of the rectum either with the intake of total dietary fibre (Fig. d), or with any of the other variables studied, including beer. This was true even when deaths from cancer of the rectum were considered in men only ($r = 0{\cdot}704$). Deaths from bronchitis and emphysema were, however, significantly correlated with beer expenditure, with vitamin D intake and SCI. The correlation with tobacco expenditure ($r = 0{\cdot}743$) was not significant at $P < 0{\cdot}01$.

TABLE III.—*Variables with which death rates are significantly correlated* ($P < 0{\cdot}001$)

| Disease | Variable | $r$ |
|---|---|---|
| Cancer of the colon | Pentose | $-0{\cdot}960$ |
| | Total vegetables excluding potatoes | $-0{\cdot}940$ |
| | Fresh green vegetables | $-0{\cdot}861$ |
| | Vitamin C | $-0{\cdot}842$ |
| | Lignin | $-0{\cdot}826$ |
| | Tobacco expenditure | $0{\cdot}770$ |
| Bronchitis and emphysema | Vitamin D | $0{\cdot}900$ |
| | SCI | $-0{\cdot}841$ |
| | Beer expenditure | $0{\cdot}841$ |

### DISCUSSION

Although the variation in nutrient intake between regions in Britain is much less than that found internationally, this study does have the advantage that uniform methods were used for assessing both diet and mortality. Other factors potentially involved in the development of cancer may also vary between regions, but there are likely to be many fewer differences in the life-style of people living within Britain than in the wide variety of countries previously included in analyses of the relationship between dietary patterns and disease (Armstrong & Doll, 1975; Drasar & Irving, 1973). The finding of a marked negative correlation between the intake of fibre pentosans or vegetables and colon cancer is therefore more likely to reflect a genuine relationship than an indirect association.

FIG.—Five-year age- and sex-standardized truncated death rates per 100,000 in relation to regional average food intake data 1969–73. Colon cancer in relation to: (a) pentose intake (g per day); (b) vegetables (excluding potatoes) (oz per week); (c) total dietary fibre (g per day); (e) fat intake (g per day); (f) beef consumption (oz per week). Rectal cancer in relation to: (d) total dietary fibre (g per day).

A possible reason for the lack of association between colon cancer and total dietary fibre is that the available food analyses for fibre may have included some starch (James & Theander, 1979). The suggestion that the pentosan intake of dietary fibre may be particularly important corresponds with the recent observation that pentosans appear to affect faecal bulking to a greater extent than other components of fibre (Cummings *et al.*, 1978).

Case-control studies have suggested that patients with large-bowel cancer have a lower consumption of vegetables than controls (Bjelke, 1970; Modan *et al.*, 1975; Graham *et al.*, 1978). These studies do not specifically relate to dietary pentosans which are particularly abundant in unrefined cereals rather than vegetables, but in Britain cereal consumption is so low that 40% of the pentosan intake comes from vegetable sources other than potatoes (potato fibre has a negligible amount of pentosan). Animal studies also suggest that some vegetables, for example the Brassicas, contain enzyme inducers which increase the metabolic capacity of polycyclic hydrocarbon hydroxylases, thus increasing the rate of metabolism of potentially carcinogenic agents (Wattenburg, 1971).

International studies on diet and colon cancer have suggested that meat, protein and fat intakes promote the development of colon cancer. Our studies do not exclude these components of the diet as risk factors, since it could be argued that the intakes in Britain are so high that the small variations observed are unlikely to affect the risk appreciably. If fat is an important dietary factor, as suggested by the relationships between fat intakes, faecal bile acid output (and concentrations) (Hill *et al.*, 1971) and colon cancer, our studies suggest that appreciable changes in intake would be needed to affect the risk, and that attempts to increase fibre intakes might be a more practicable way of reducing the incidence of colon cancer.

The regional pattern of death rates from colon cancer is similar to that of almost all diseases in Great Britain, with higher rates in the North and West than in the South and East. Associations could reflect a coincidence in the geography of both diet and disease. However, statistical analysis did not reveal a significant association between fibre pentosan intake or vegetable consumption and mortality from bronchitis ($r = -0{\cdot}387$ and $-0{\cdot}453$) but nevertheless, the limitations of an analysis such as this must be recognized. There is no support from other studies for the association of bronchitis death rates with vitamin D and beer consumption, and they probably result from the interrelationship between social class, beer drinking and the consumption of foods high in vitamin D, such as margarine. No relationship was found between colon-cancer mortality and social class, in agreement with other analyses (Office of Population Censuses and Surveys, 1978).

We chose to look at regional patterns of mortality, rather than incidence, as comprehensive incidence data for areas comparable with those used by the household surveys are not available. Regional differences in survival from colon cancer may be present, the relationship to vegetable intake then reflecting the propensity of regions to be more health conscious, with earlier and better treatment.

Despite these provisos, however, these results do suggest the need for further work designed to test whether specific dietary fibre components or vegetables protect from carcinogenesis within the colon. Intakes of dietary fibre components and their sources now need to be obtained from case-control studies and regional studies in countries with a greater variability of consumption combined with satisfactory data on the incidence of large-bowel cancer.

The authors gratefully acknowledge the help of Dr D. A. T. Southgate who made available many unpublished analyses and for his advice in the preparation of the dietary fibre file, Miss J. Robertson, M.A.F.F., for unpublished data from the N.F.S., Mr L. Bulusu, Division of Medical Statistics, O.P.C.S., who kindly made the data on deaths available to us prior to publication, and Mr P. Mattock, D.O.E., for regional ratios of household expenditure of beer and cider relative to the U.K. D.R.R.W. is in receipt of an MRC Training Fellowship in Epidemiology.

### REFERENCES

ARMSTRONG, B. & DOLL, R. (1975) Environmental factors and cancer incidence in different countries, with special reference to dietary practices. *Int. J. Cancer*, **15**, 617.

BJELKE, E. (1970) Case control study of cancer of the stomach, colon and rectum. In *Oncology* 1970. Eds. R. L. Clark, R. W. Cumley, J. E. McCay & M. M. Copeland. Proc. 10th Int. Cancer Cong. Vol. V. Chicago, Ill: Year Book Medical Publishers Inc. 1971. p. 320.

BURKITT, D. P., WALKER, A. R. P. & PAINTER, N. S. (1972) Effect of dietary fibre on stools, and transit time and its role in causation of disease. *Lancet*, ii, 1408.

CUMMINGS, J. H., BRANCH, W. J., JENKINS, D. J. A., SOUTHGATE, D. A. T., HOUSTON, H. & JAMES, W. P. T. (1978) Colonic response to dietary fibre from carrot, cabbage, apple, bran and guar gum. *Lancet*, i, 5.

DEPARTMENT OF EMPLOYMENT (1970–74) *Family Expenditure Surveys* 1969–73. London: H.M.S.O.

DRASAR, B. S. & IRVING, D. (1973) Environmental factors and cancer of the colon and breast. *Br. J. Cancer*, **27**, 167.

ENSTROM, J. E. (1977) Colorectal cancer and beer drinking. *Br. J. Cancer*, **35**, 674.

Graham, S., Dayal, H., Swanson, M., Mittelman, A. & Wilkinson, G. (1978) Diet in the epidemiology of cancer of the colon and rectum. *J. Natl Cancer Inst.*, **61**, 709.

Gregor, O., Toman, R. & Prusova, F. (1969) Gastrointestinal cancer and nutrition. *Gut*, **10**, 1031.

Haenszel, W., Berg, J. W., Segi, M., Kurihara, M. & Locke, F. B. (1973) Bowel cancer in Hawaiian Japanese. *J. Natl Cancer Inst.*, **51**,1765.

Haenszel, W. & Kurihara, M. (1968) Studies of Japanese migrants mortality from cancer and other diseases among Japanese in the United States. *J. Natl Cancer Inst.*, **40**, 43.

Heasman, M. A. & Lipworth, L. (1966) *Accuracy of Certification of Cause of Death*. London: H.M.S.O.

Hill, M. J., Crowther, J. S., Drasar, B. S., Hawkesworth, G., Aries, V. & Williams, R. E. O. (1971) Bacteria and aetiology of cancer of large bowel. *Lancet*, i, 95.

I.A.R.C. Intestinal Microecology Group (1977) Dietary fibre, transit time, faecal bacteria, steroids and colon cancer in two Scandinavian populations. *Lancet*, ii, 207.

James, W. P. T., Branch, W. J. & Southgate, D. A. T. (1978) Calcium binding by dietary fibre. *Lancet*, i, 638.

James, W. P. T. & Theander, O. (1979) (Eds) *Report of an E.E.C. working party on the analysis of dietary fibre*. New York: Marcel Dekker (in press).

McCance, R. A. & Widdowson, E. M. (1967) The composition of foods. *Med. Res. Council Spec. Rep. Ser.*, **297**. London: HMSO.

Ministry of Agriculture, Fisheries and Food (1971–75) Household food consumption and expenditure 1969–73. *Ann. Rep. Natl Food Survey Committee*. London: HMSO.

Modan, B., Barell, V., Lubin, F., Modan, M., Greenberg, R. & Graham, S. (1975) Low-fiber intake as an etiologic factor in cancer of the colon. *J. Natl Cancer Inst.*, **55**, 15.

Office of Population Censuses and Surveys. *Census* 1971 *Great Britain, Economic Activity Part IV* (10% *sample*). p. 184.

Office of Population Censuses and Surveys (1978) *Occupational Mortality D.S. No.* 1. *The Registrar General's Decennial Supplement for England and Wales* 1970–72.

Registrar General for Scotland, *Annual Reports* 1969–1973. Table C.2.1.

Southgate, D. A. T. (1978) Dietary fibre: analysis and food sources. *Am. J. Clin. Nutr. Supple.*, **31**, S107.

Southgate, D. A. T., Bailey, B., Collinson, E. & Walker, A. F. (1976) A guide to calculating intakes of dietary fibre. *J. Hum. Nutr.*, **30**, 303.

Southgate, D. A. T., Bingham, S. & Robertson, J. (1978) Dietary fibre in British diet. *Nature*, **274**, 51.

Van Soest, P. J. & Robertson, J. B. (1976) What is fibre and fibre in food? *Nutr. Rev.*, **35**, 13.

Wattenburg, L. W. (1971) Studies on the polycyclic hydrocarbon hydroxylases of the intestine possibly related to cancer. *Cancer*, **28**, 99.

World Health Organisation (1964) Prevention of cancer. *Tech. Rep. Ser.*, **276**.